# Supplementary material for: Differential chloride homeostasis in the spinal dorsal horn locally shapes synaptic metaplasticity and modality-specific sensitization
Source: Nat Commun. 2020 Aug 7;11:3935. doi: 10.1038/s41467-020-17824-y (PMC7414850; doi:10.1038/s41467-020-17824-y)
Supplement: Supplementary file 1 — Supplementary Information [file 41467_2020_17824_MOESM1_ESM.pdf]

Supplementary Information

Differential chloride homeostasis in the spinal dorsal horn locally shapes synaptic metaplasticity and modality-specific sensitization

Ferrini et al.

Nature Communications 2020

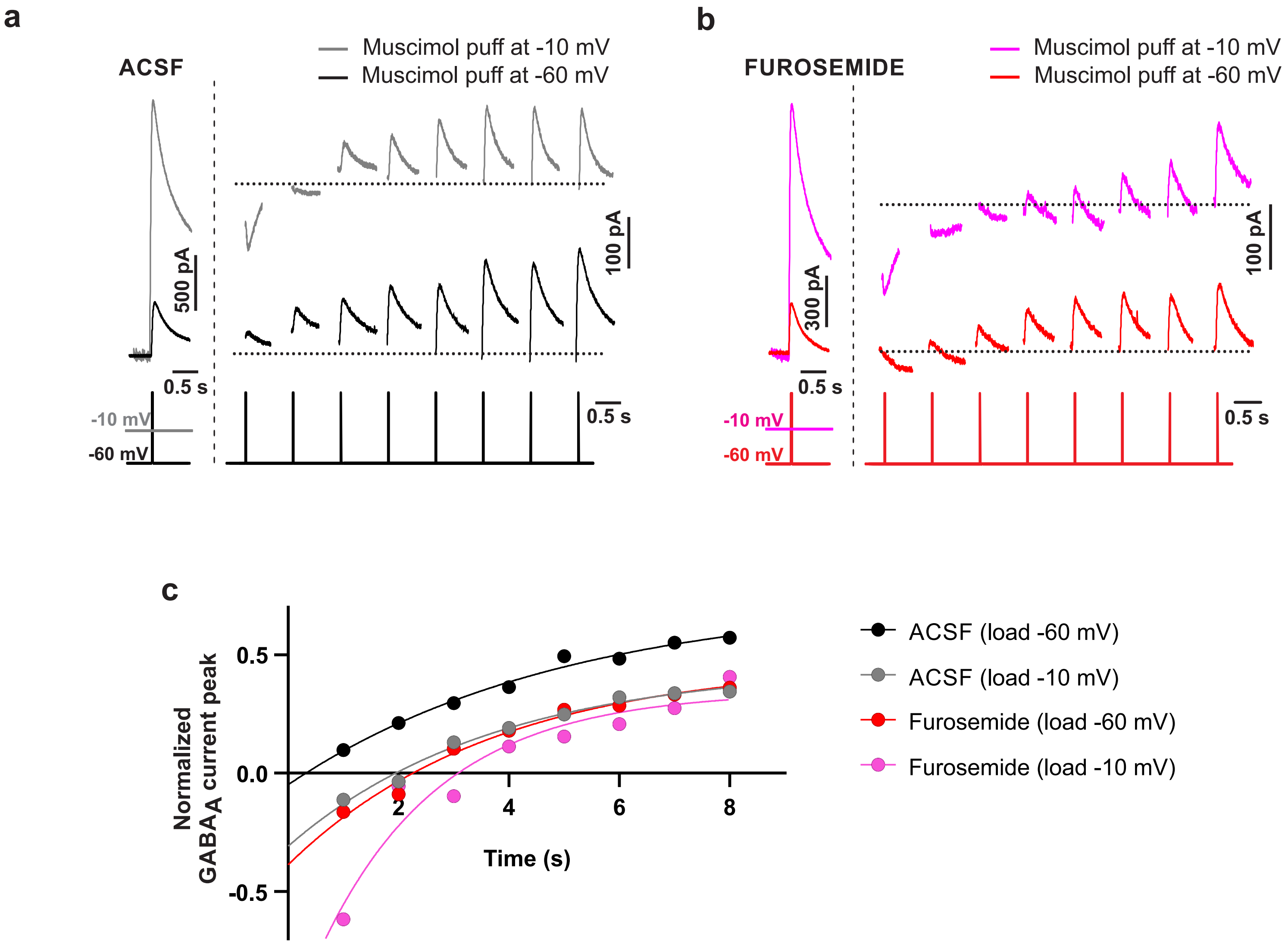

Supplementary Figure 1. Role of KCC2 in maintaining gradient following a large muscimol-induced conditioning current. (a) $GABA_A$ currents evoked by puffing muscimol (500 μM). Currents were recorded with a low Cl^- (9 mM) pipette solution at regular intervals either following an initial *test stimulus* (muscimol puff) delivered at -60 mV (*black*, Cl^- driving force is small and Cl^- influx modest), or following a *conditioning stimulus* (muscimol puff) at -10 mV (*grey*, Cl^- driving force is large and Cl^- influx higher). The polarity of $GABA_A$ currents was measured at increasing intervals, equally spaced by 1 s, following the *test*/*conditioning stimulus*, for a total of 8 pulses. Each pair of *test*/*conditioning stimulu*s and $GABA_A$ current was delivered in subsequent trials with 30 s inter-trial interval. The test pulse at -60 mV does not affect the polarity of subsequent $GABA_A$ currents; conversely, the first $GABA_A$ current recorded at -60 mV after a large Cl^- load at -10 mV is inwardly directed. Polarity is quickly restored within 2s. (b) In presence of furosemide (100 μM), even the relatively small Cl^- influx induced by test stimulus at -60 mV affects the polarity of the subsequent $GABA_A$ current (*red*). The effect is more dramatic after the conditioning stimulus at 0 mV, after which $GABA_A$ current is inward for the subsequent 3 seconds and biphasic for additional 2s, indicating a reduced capacity to restore intracellular Cl^- concentration. (c) Time course of $GABA_A$ current peak amplitude (normalized to the *test stimulus*) following the *conditioning stimulus*. Abbreviation: ACSF = artificial cerebro-spinal fluid.

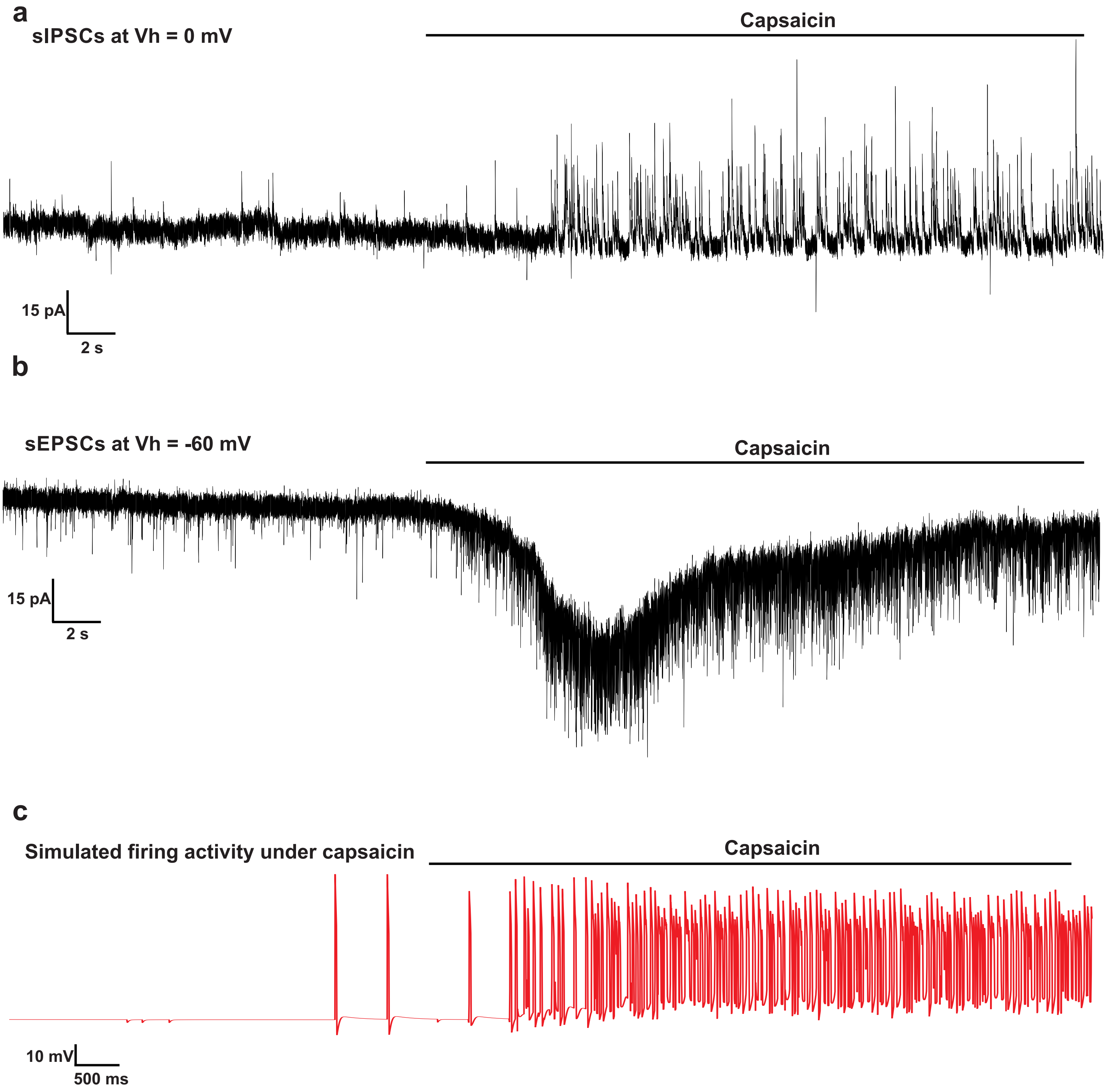

Supplementary Figure 2. Increased synaptic activity and firing after capsaicin administration. (**a-b**) Capsaicin administration (2 μM) increased both spontaneous inhibitory post-synaptic currents recorded at 0 mV (**a**) and excitatory currents at -60 mV (**b**). (**c**) Sample trace of membrane potential simulated in silico by an electrodiffusion model before and after capsaicin administration. Abbreviations: sIPSCs = spontaneous inhibitory post-synaptic currents, sEPSCs = spontaneous excitatory post-synaptic currents.

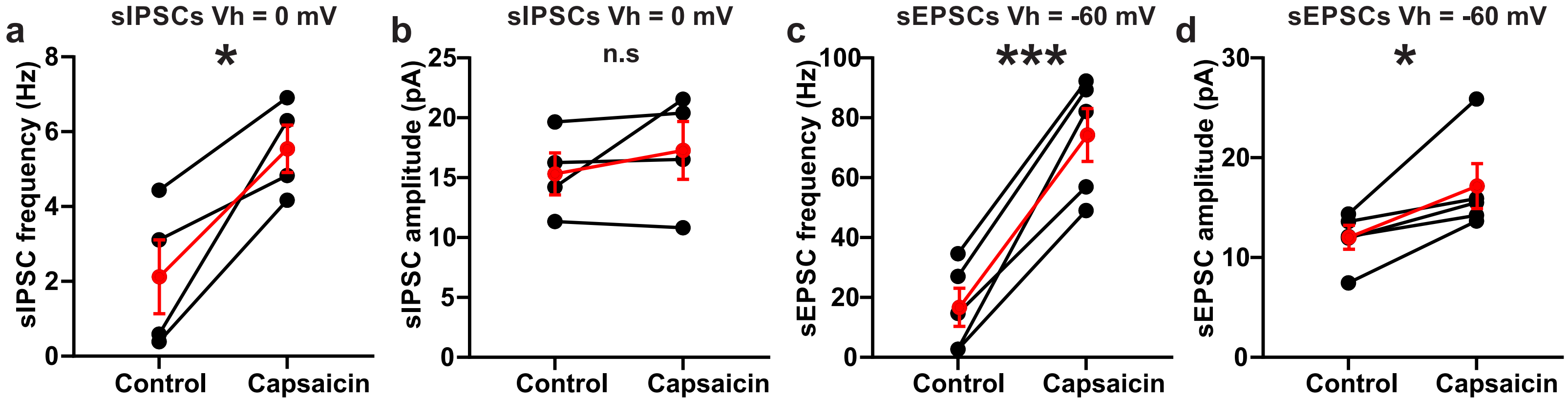

Supplementary Figure 3. sIPSC and sEPSC frequency and amplitude after capsaicin treatment. (**a-b**) sIPSC frequency (**a**, n = 4, paired t-test, *P* = 0.03) and amplitude (**b**, n = 4, paired t-test, *P* = 0.36) before and after bath-applied capsaicin (2 μM). (**c-d**) sEPSC frequency (**a**, n = 5, paired t-test, *P* < 0.001) and amplitude (**b**, n = 5, paired t-test, *P* = 0.04) before and after bath-applied capsaicin (2 μM). Abbreviations: sIPSCs = spontaneous inhibitory post-synaptic currents, sEPSCs = spontaneous excitatory post-synaptic currents, n.s. = not significant. Data are shown as mean ± S.E.M. *P < 0.05, ***P < 0.001.

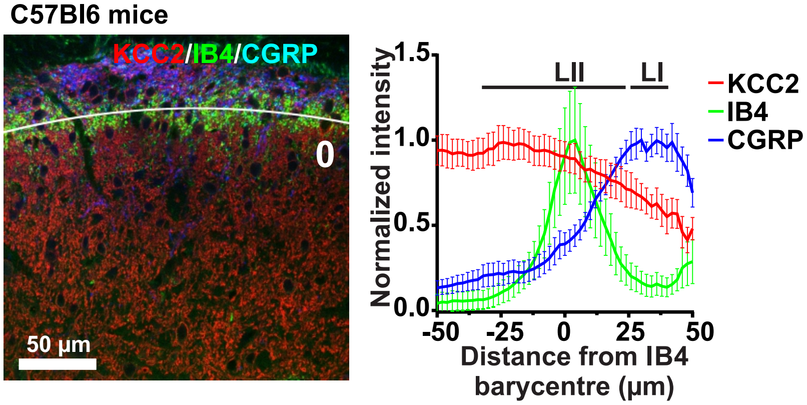

Supplementary Figure 4. **Interlaminar gradient of KCC2 in the mouse superficial spinal dorsal horn**. Triple fluorescence staining for CGRP (*blue*), IB4 (*green*) and KCC2 (*red*) in the mouse SDH. The *white line* represents the barycentric origin (0) of IB4 staining. In the histogram, CGRP, IB4 and KCC2 mean fluorescence intensities in the SDH are reported as a function of the position towards the IB4 barycentre (lamina I average fluorescence intensity 411 ± 57 i.u., lamina II 581 ± 60 i.u., n = 8, paired t-test $P < 0.001$). Abbreviations: i.u. = intensity unit, CGRP = calcitonin gene-related peptide, IB4 = isolectin B4. Data are shown as mean ± S.E.M.

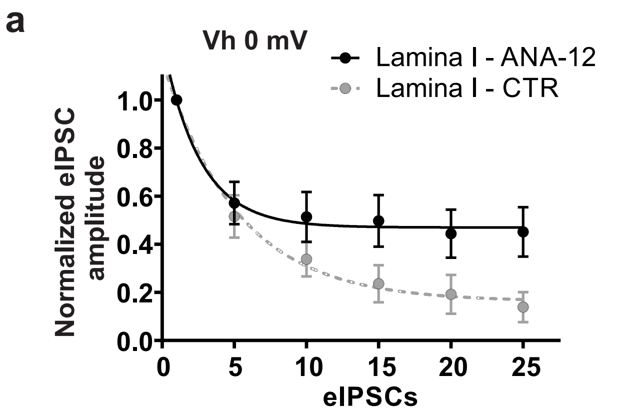

Supplementary Figure 5. Enhancing KCC2 prevents activity-dependent collapse of inhibition in lamina I. (**a**) Depression of eIPSC amplitudes in lamina I during 20 Hz repetitive stimulation following ANA-12 treatment (1 μM, 2 hours – n = 6, *solid line*). For comparison, the lamina I depression in control (Fig. 6b) is also shown (*dashed line*). (**b**) Depression of eIPSC amplitudes in lamina I during 20 Hz repetitive stimualtion following CLP257 treatment (5 μM, 2 hours – n = 5, *solid line*). For comparison, the lamina I depression in control (Fig. 6b) is also shown (*dashed line*). Abbreviations: ANA-12 = N-[2-[[(Hexahydro-2-oxo-1H-azepin-3-yl)amino]carbonyl]phenyl]benzo[b]thiophene-2-carboxamide, eIPSCs = evoked inhibitory post-synaptic currents. Data are shown as mean ± S.E.M.

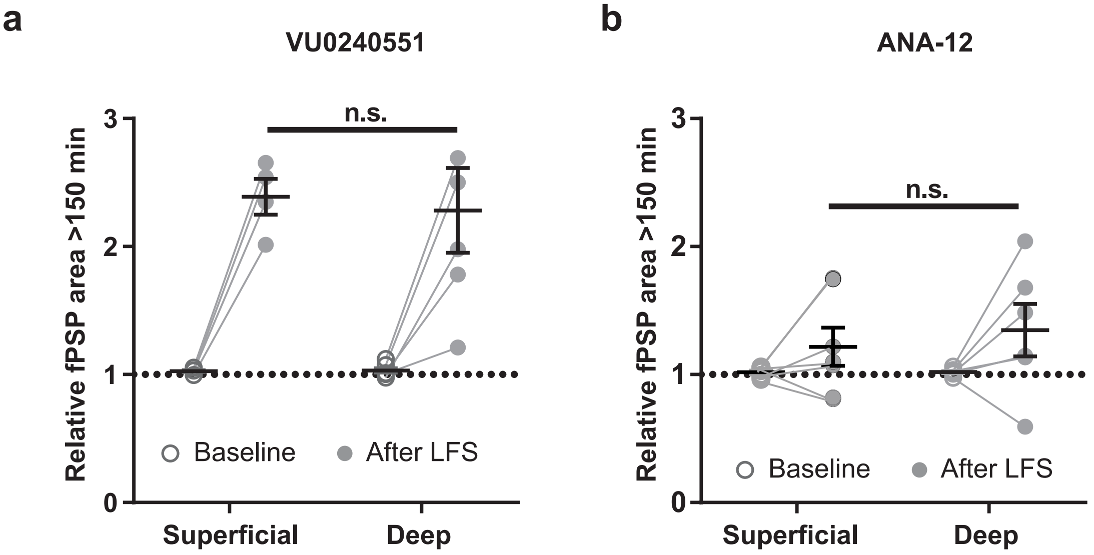

Supplementary Figure 6. **Differences in LTF in superficial and deep recordings were abolished by VU0240551 and ANA-12 treatments.** (**a**) Low frequency stimulation (LFS) produced similar *runaway* increase in fPSP responses in superficial (n = 5) and deep recordings (n = 5) after blocking KCC2 activity with VU0240551 (10 µM; Two-way-RM-ANOVA, $F_{interaction}$ = 0.1, P = 0.8). (**b**) ANA-12 (1 µM) administration produced similar increase of fPSP responses in superficial (n = 7) and deep (n = 6; Two-way-RM-ANOVA, $F_{interaction}$ = 0.3, P = 0.6) recordings 150 min after LFS. Individual experiments are depicted in grey. Abbreviations: n.s. = not significant. Data are shown as mean ± S.E.M.

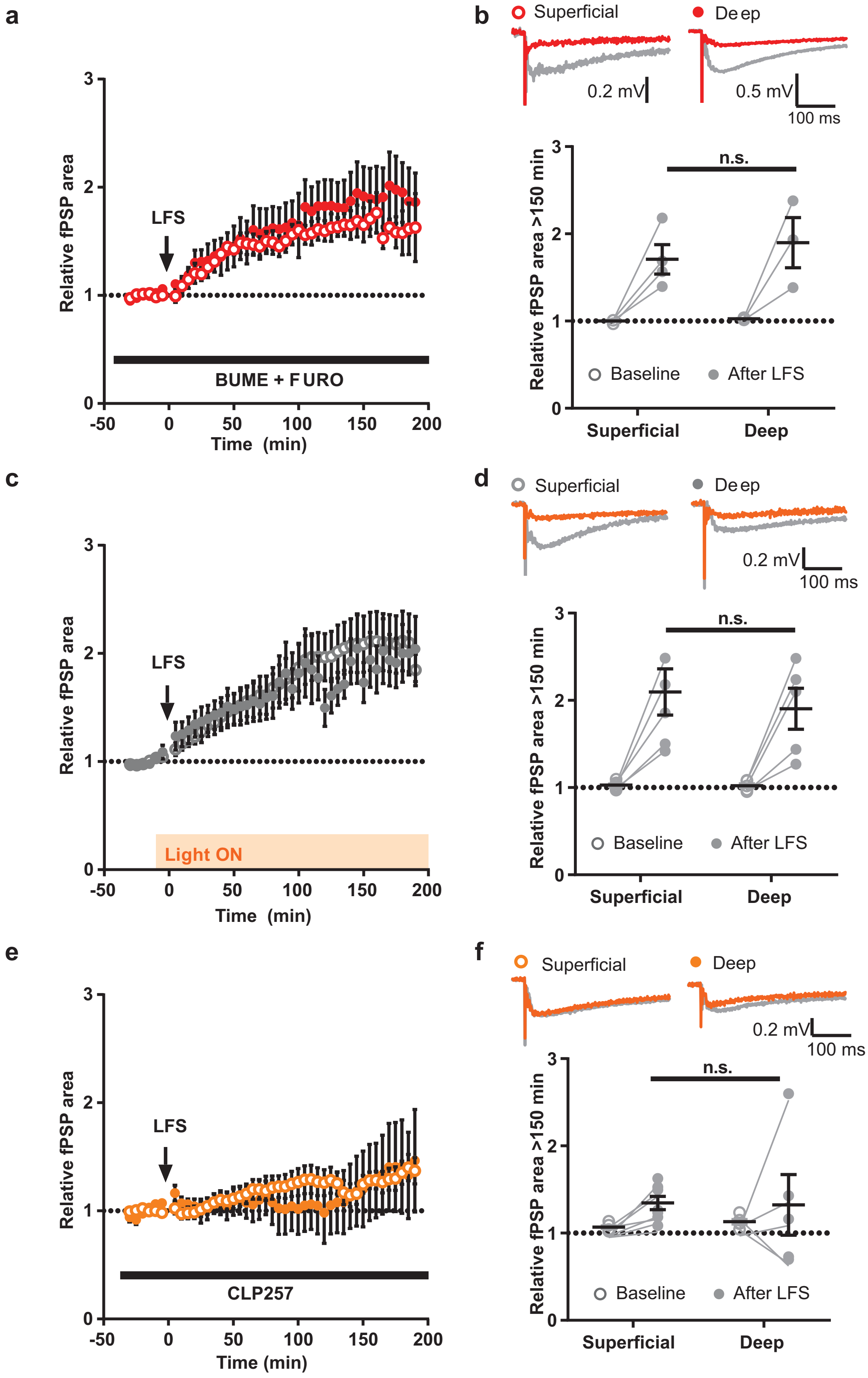

Supplementary Figure 7. Increased chloride loading in lamina I leads to an unconstrained LTF. (**a**) LFS-induced increase of fPSP responses in superficial and deep recordings with furosemide (100 µM) treatment in explants continuously treated with bumetanide (10 µM; Two-way-RM-ANOVA $F_{interaction}$ = 0.5, P = 0.9). Arrow shows the time of LFS application. (**b**) LFS produced a similar increase in fPSP responses in superficial (n = 4) and deep (n = 3) recordings after 150 min under bumetanide and furosemide administration (Two-way-RM-ANOVA, $F_{interaction}$ = 0.3, *P* = 0.6). Insets on top show representative traces of fPSP responses during baseline (*red*) and 150 min after LFS (*grey*). (**c**) LFS-induced increase of fPSP responses in superficial and deep recordings with Cl^- loading in spinal cord neurons by optogenetic-activation of the chloride-pump NpHR3.0 (Two-way-RM-ANOVA, $F_{interaction}$ = 0.8, *P* = 0.6). (**d**) NpHR3.0-induced Cl^- loading produced similar increase in fPSP responses in superficial (n = 6) and deep (n = 5) recordings after 150 min of LFS (Two-way-RM-ANOVA, $F_{interaction}$ = 0.3, *P* = 0.6). Representative traces are shown on top. (**e**) LFS-induced increase of fPSP responses in superficial and deep recordings with CLP257 (5 µM) administration (Two-way-RM-ANOVA, $F_{interaction}$ = 0.6, *P* = 0.9). (**f**) CLP257 decreased synaptic plasticity in superficial recordings (n = 7), similar to deep recordings (n = 5) 150 min of LFS (Two-way-RM-ANOVA, $F_{interaction}$ = 0.1, *P* = 0.8). Representative traces are shown on top. Individual experiments are depicted in grey. Abbrevaitions: LFS = low frequency stimulation, n.s. = not significant. Data are shown as mean ± S.E.M.

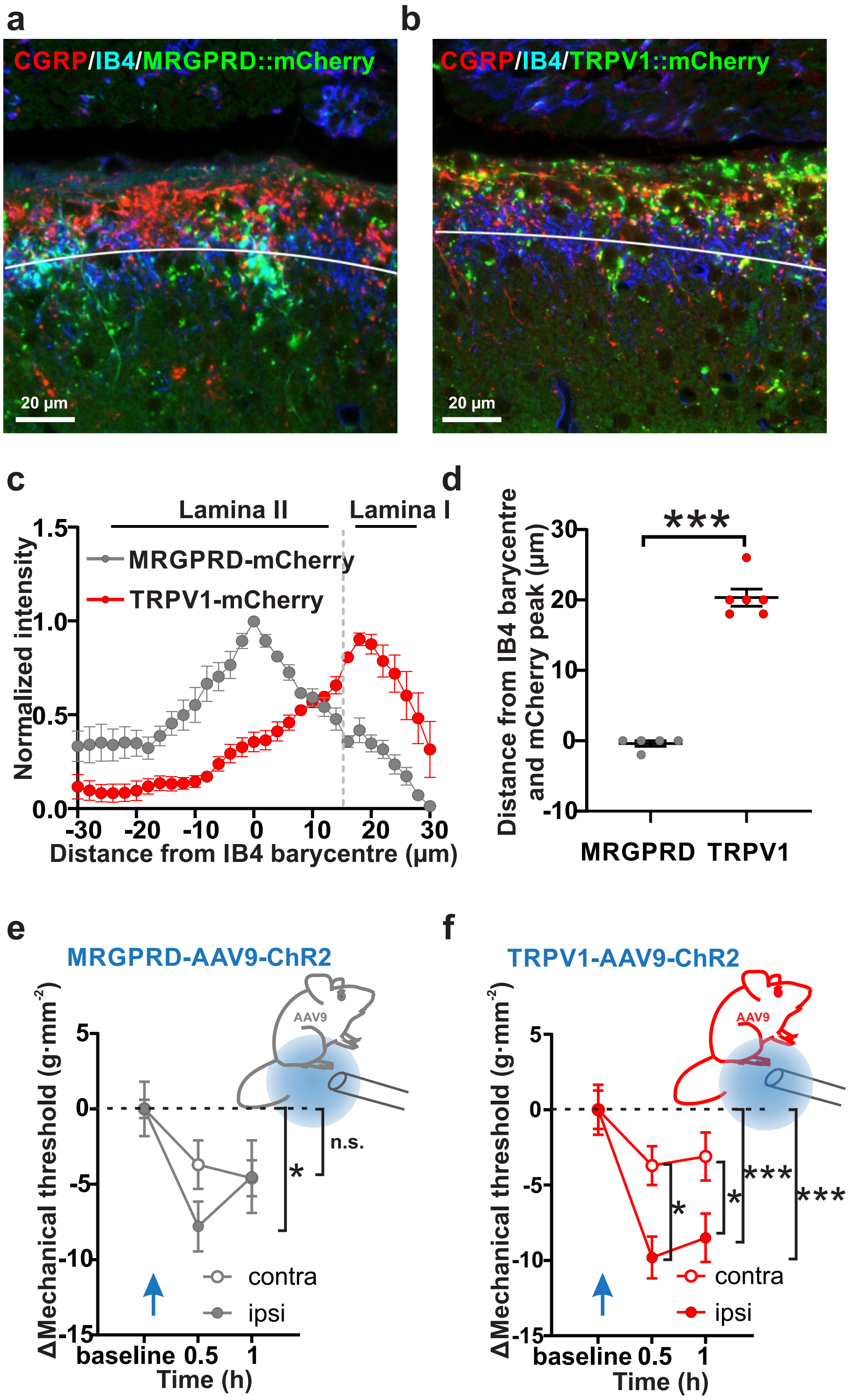

Supplementary Figure 8. Modality-specific nociceptive processing assessed by viral delivery of Cre-dependent ChR2. (**a-b**) Confocal images of the distribution of postnatal mCherry+ projections in the superficial dorsal horn of AAV9-FLEX-rev-ChR2(H134R)-mCherry-injected MRGPRD-Cre (**a**) and TRPV1-Cre (**b**) mice. Laminar boundaries were determined by CGRP (*red*) and IB4 (*blue*) staining. The *white line* represents the IB4 barycentre in the SDH. (**c**) Distribution of mCherry+ fluorescence intensity from 5 MRGPRD-ChR2 and 6 TRPV1-ChR2 as a function of the IB4 barycentre (*white line*). Dashed line indicates the boundary between lamina I and II. (**d**) The peak of mCherry-intensity associated to MRGPRD afferents (*grey*) is closer to the IB4 barycentre (lamina II) than TRPV1 afferents (*red;* t-test, P< 0.001). (**e-f**) Time course of mechanical sensitization induced by blue light illumination (5 min, 2 Hz) of the hind paw in MRGPRD-ChR2 (**e**, n = 6; Two-way-RM-ANOVA, F_{time} = 6.0, *P* = 0.007) and TRPV1-ChR2 mice (**f**, n = 9; Two-way-RM-ANOVA, F_{time} = 12.0, *P* < 0.001). The insets schematically illustrate the experimental procedure. Abbreviations: i.u. = intensity units, CGRP = calcitonin gene-related peptide, IB4 = isolectin B4, MRGPRD = mas-related G-protein coupled receptor member D, TRPV1 = Transient receptor potential vanilloid 1, ChR2 = channelrhodopsin 2, AAV9 = adeno-associated virus serotype 9, contra = contralateral, ipsi = ipsilateral. Data are expressed as mean ± S.E.M. *P < 0.05, ***P < 0.001.
