# Supplementary material for: Differential chloride homeostasis in the spinal dorsal horn locally shapes synaptic metaplasticity and modality-specific sensitization
Source: Nat Commun. 2020 Aug 7;11:3935. doi: 10.1038/s41467-020-17824-y (PMC7414850; doi:10.1038/s41467-020-17824-y)
Supplement: Supplementary file 3 — Reporting Summary [file 41467_2020_17824_MOESM3_ESM.pdf]

nature research

Corresponding author(s): Francesco Ferrini

Last updated by author(s): Jul 15, 2020

Reporting Summary

Nature Research wishes to improve the reproducibility of the work that we publish. This form provides structure for consistency and transparency in reporting. For further information on Nature Research policies, see Authors & Referees and the Editorial Policy Checklist .

Statistics

For all statistical analyses, confirm that the following items are present in the figure legend, table legend, main text, or Methods section.

n/a	Confirmed	
☐	☒	The exact sample size (*n*) for each experimental group/condition, given as a discrete number and unit of measurement
☐	☒	A statement on whether measurements were taken from distinct samples or whether the same sample was measured repeatedly
☐	☒	The statistical test(s) used AND whether they are one- or two-sided<br>*Only common tests should be described solely by name; describe more complex techniques in the Methods section.*
☒	☐	A description of all covariates tested
☐	☒	A description of any assumptions or corrections, such as tests of normality and adjustment for multiple comparisons
☐	☒	A full description of the statistical parameters including central tendency (e.g. means) or other basic estimates (e.g. regression coefficient) AND variation (e.g. standard deviation) or associated estimates of uncertainty (e.g. confidence intervals)
☐	☒	For null hypothesis testing, the test statistic (e.g. *F*, *t*, *r*) with confidence intervals, effect sizes, degrees of freedom and *P* value noted<br>*Give P values as exact values whenever suitable.*
☒	☐	For Bayesian analysis, information on the choice of priors and Markov chain Monte Carlo settings
☒	☐	For hierarchical and complex designs, identification of the appropriate level for tests and full reporting of outcomes
☒	☐	Estimates of effect sizes (e.g. Cohen's *d*, Pearson's *r*), indicating how they were calculated

Our web collection on statistics for biologists contains articles on many of the points above.

Software and code

Policy information about availability of computer code

Data collection	COMSOL Multiphysics environment (http://www.comsol.com), WinWCP (v.4.5.0, Dr. J. Dempster, University of Strathclyde, Glasgow, UK), pClamp 10.2 software (Molecular Devices)
Data analysis	GraphPad Prism 8 (GraphPad Software), MATLAB software (The MathWorks Inc), Clampfit 10.2 (Molecular Devices), Image J 1.47h (NIH), MiniAnalysis Software (Synaptosoft).

For manuscripts utilizing custom algorithms or software that are central to the research but not yet described in published literature, software must be made available to editors/reviewers. We strongly encourage code deposition in a community repository (e.g. GitHub). See the Nature Research guidelines for submitting code & software for further information.

Data

Policy information about availability of data

All manuscripts must include a data availability statement. This statement should provide the following information, where applicable:

- Accession codes, unique identifiers, or web links for publicly available datasets
- A list of figures that have associated raw data
- A description of any restrictions on data availability

All data supporting the findings of this study are available within the article or from the corresponding author upon reasonable request

Field-specific reporting

Please select the one below that is the best fit for your research. If you are not sure, read the appropriate sections before making your selection.

☒ Life sciences ☐ Behavioural & social sciences ☐ Ecological, evolutionary & environmental sciences

Life sciences study design

All studies must disclose on these points even when the disclosure is negative.

Sample size	No specific statistical tests were used to determine sample size. Sample sizes were chosen based on available information on variability from previous electrophysiological, behavioral and immunohistochemical datasets.
Data exclusions	No data were excluded from the study, except a single data point identified as outlier by ROUT (Robust regression and Outlier removal, Q = 1%) in Fig. 6d (y = 4.8) was removed. Specific Exclusion criteria for electrphysiological data are reported in methods.
Replication	All attempts to replicate were successful. Key experimental findings were independently confirmed by using different approaches performed by different operators (patch clamp, immunostaining, chloride imaging, field recordings, and optogenetics).
Randomization	Samples/animals were randomly allocated into groups.
Blinding	Quantifications on treated groups vs controls were performed by operators unaware of the treatment

Reporting for specific materials, systems and methods

We require information from authors about some types of materials, experimental systems and methods used in many studies. Here, indicate whether each material, system or method listed is relevant to your study. If you are not sure if a list item applies to your research, read the appropriate section before selecting a response.

Materials & experimental systems

n/a	Involved in the study	
☐	☒	Antibodies
☒	☐	Eukaryotic cell lines
☒	☐	Palaeontology
☐	☒	Animals and other organisms
☒	☐	Human research participants
☒	☐	Clinical data

Methods

n/a	Involved in the study	
☒	☐	ChIP-seq
☒	☐	Flow cytometry
☒	☐	MRI-based neuroimaging

Antibodies

Antibodies used	Rabbit anti-KCC2 (Millipore-Upstate, CAT# 07-432; RRID: AB_310611), mouse anti-CGRP (Sigma catalog CAT# C7113; RRID: AB_259000), chicken anti-full lenght-TrkB antibody (Promega Corporation, Cat# G1561, RRID: AB_430846 lot#127175), rabbit polyclonal anti-NK1 (Sigma-Aldrich, CAT# SAB4502913; RRID: AB_10746598), rabbit polyclonal anti-PKCγ (CAT# SC-211; RRID: AB_632234).
Validation	All antibodies were validated in previous studies: rabbit anti-KCC2 by Wiliams et al. Journal of Biological Chemistry 1999; mouse anti-CGRP by Magnussen et al., Molecular Pain, 2015; chicken anti-full lenght-TrkB by Salio et al. European Journal of Neuroscience 2005; rabbit anti-NK1 by Peirs et. al, Neuron 2015; rabbit anti-PKCγ by Abraira et al., Cell 2017. Negative controls were routinely performed in our study.

Animals and other organisms

Policy information about studies involving animals; ARRIVE guidelines recommended for reporting animal research

Laboratory animals	Male Sprague Dawley rats (>60 days), male C57Bl/6 mice (>60 days) and transgenic mice (Ai39 Jackson Laboratory #014539; MMRRC, stock No. 036118-UNC x Ai32 Jackson Laboratory #012569; Jackson Laboratory #017769 x Ai32 Jackson Laboratory #012569; Jackson Laboratory #010802 x Ai32 Jackson Laboratory #012569 lines) (>60 days).
Wild animals	No wild animals were used in the study.
Field-collected samples	No samples were collected in the field.
Ethics oversight	All experimental procedures have been performed in accordance with guidelines from the Canadian Council on Animal Care and approved by the committee for animal protection of Université Laval (CPAUL; authorization number: 2018-027-1 and 2018-026-1).

Note that full information on the approval of the study protocol must also be provided in the manuscript.
